# Psychometric Properties of a Machine Learning–Based Patient-Reported Outcome Measure on Medication Adherence: Single-Center, Cross-Sectional, Observational Study

**DOI:** 10.2196/42384

**Published:** 2023-10-16

**Authors:** Virginie Korb-Savoldelli, Yohann Tran, Germain Perrin, Justine Touchard, Jean Pastre, Adrien Borowik, Corine Schwartz, Aymeric Chastel, Eric Thervet, Michel Azizi, Laurence Amar, Benjamin Kably, Armelle Arnoux, Brigitte Sabatier

**Affiliations:** 1 Pharmacy Department Hôpital européen Georges Pompidou, Assistance Publique - Hôpitaux de Paris (APHP) Paris Cedex 15 France; 2 Clinical Pharmacy Department Faculty of Pharmacy Paris-Saclay University Orsay France; 3 Clinical Research Unit Université Paris Cité Hôpital européen Georges Pompidou, Assistance Publique - Hôpitaux de Paris (APHP) Paris France; 4 Clinical Investigation Center (CIC) 1418 Clinical Epidemiology Institut National de la Santé et de la Recherche Médicale (INSERM) Hôpital européen Georges Pompidou, Assistance Publique-Hôpitaux de Paris (APHP) Paris France; 5 Health data- and model- driven Knowledge Acquisition (HeKA) Team Institut National de la Santé et de la Recherche Médicale (INSERM) - (Institut National de Recherche en Informatique et en Automatique (INRIA) PariSanté Campus Paris France; 6 Pulmonary Medecine and Intensive Care Department Hôpital européen Georges Pompidou Assistance Publique - Hôpitaux de Paris (APHP) Paris France; 7 Nephrology Department Hôpital européen Georges Pompidou Assistance Publique - Hôpitaux de Paris (APHP) Paris France; 8 Institut National de la Santé et de la Recherche Médicale (INSERM) - Unité Mixte de Recherche (UMR) 970 – Team 8, Paris Cardiovascular Research Center (PARCC) Hôpital européen Georges Pompidou Assistance Publique-Hôpitaux de Paris (APHP) Paris France; 9 Hypertension Department Reference Centre for Rare Vascular Disease Hôpital européen Georges Pompidou, Assistance Publique - Hôpitaux de Paris (APHP) Paris France; 10 Pharmacology Unit Hôpital Européen Georges Pompidou Assistance Publique - Hôpitaux de Paris (APHP) Paris France

**Keywords:** medication adherence, long-term therapies, machine learning, patient-reported outcome measure, decision tree, predict, Delphi, cross sectional, psychometric, mobile phone

## Abstract

**Background:**

Medication adherence plays a critical role in controlling the evolution of chronic disease, as low medication adherence may lead to worse health outcomes, higher mortality, and morbidity. Assessment of their patients' medication adherence by clinicians is essential for avoiding inappropriate therapeutic intensification, associated health care expenditures, and the inappropriate inclusion of patients in time- and resource-consuming educational interventions. In both research and clinical practices the most extensively used measures of medication adherence are patient-reported outcome measures (PROMs), because of their ability to capture subjective dimensions of nonadherence. Machine learning (ML), a subfield of artificial intelligence, uses computer algorithms that automatically improve through experience. In this context, ML tools could efficiently model the complexity of and interactions between multiple patient behaviors that lead to medication adherence.

**Objective:**

This study aimed to create and validate a PROM on medication adherence interpreted using an ML approach.

**Methods:**

This cross-sectional, single-center, observational study was carried out a French teaching hospital between 2021 and 2022. Eligible patients must have had at least 1 long-term treatment, medication adherence evaluation other than a questionnaire, the ability to read or understand French, an age older than 18 years, and provided their nonopposition. Included adults responded to an initial version of the PROM composed of 11 items, each item being presented using a 4-point Likert scale. The initial set of items was obtained using a Delphi consensus process. Patients were classified as poorly, moderately, or highly adherent based on the results of a medication adherence assessment standard used in the daily practice of each outpatient unit. An ML-derived decision tree was built by combining the medication adherence status and PROM responses. Sensitivity, specificity, positive and negative predictive values (NPVs), and global accuracy of the final 5-item PROM were evaluated.

**Results:**

We created an initial 11-item PROM with a 4-point Likert scale using the Delphi process. After item reduction, a decision tree derived from 218 patients including data obtained from the final 5-item PROM allowed patient classification into poorly, moderately, or highly adherent based on item responses. The psychometric properties were 78% (95% CI 40%-96%) sensitivity, 71% (95% CI 53%-85%) specificity, 41% (95% CI 19%-67%) positive predictive values, 93% (95% CI 74%-99%) NPV, and 70% (95% CI 55%-83%) accuracy.

**Conclusions:**

We developed a medication adherence tool based on ML with an excellent NPV. This could allow prioritization processes to avoid referring highly adherent patients to time- and resource-consuming interventions. The decision tree can be easily implemented in computerized prescriber order-entry systems and digital tools in smartphones. External validation of this tool in a study including a larger number of patients with diseases associated with low medication adherence is required to confirm its use in analyzing and assessing the complexity of medication adherence.

## Introduction

Medication adherence is defined as the extent to which patients take medications as prescribed by their physicians [1]. Medication adherence plays a key role in controlling chronic disease evolution, as these conditions require continuous long-term treatment [2]. Low medication adherence may lead to worse health outcomes and higher mortality and morbidity [3-5]. This long-standing problem is growing with improvements in medication-based management of chronic diseases, for which patients do not always perceive symptoms [6,7]. According to the World Health Organization (WHO), only half of the patients worldwide adhere to their prescribed medication regimen [2]. The reasons for nonadherence identified by the WHO are related to the disease, treatments, patients, and relationship between patients and health care providers [2]. Improving medication adherence is a cornerstone for improving treatment outcomes. Nonadherence also incurs a high cost burden to health care systems by increasing hospital visits, as well as causing unnecessary escalation to more costly treatments. According to an European Union report, therapy nonadherence is responsible for 194,500 deaths annually, costing €125 (US $135) billion [8]. Despite the contribution of medication nonadherence in the worsening of disease and increased health care costs, many clinicians are not properly trained to screen for it [9]. Identifying adherence‐related behaviors of patients with chronic diseases is an important step toward improving medication adherence and patient education [10]. To achieve this goal, numerous direct and indirect tools have been proposed [11]. As medication adherence is a complex multifactorial behavior, it is important to propose accurate and easy-to-use tools for measuring medication adherence in daily medical practice to understand patient behavior toward medications. In this context, the use of patient-reported outcome measures (PROMs) is of great interest, as these self-reporting tools are able to address the problem of assessing subjective information about patients, such as their health priorities, experience, and perception of outcome, which are major aspects that explain medication nonadherence [12]. Data obtained using PROMs directly arise from the patient, without the involvement of the clinician’s interpretation. Thus, PROMs are relevant tools to drive health care decision-making processes [13]. Medication adherence is an example of subjective behavior that can be captured using PROMs in clinical practice and research to guide decisions concerning the support and treatment benefits of medication adherence or harm of nonadherence [12]. PROMs are the most extensively used measures of medication adherence, in both research and clinical settings, for several reasons: they are easy to administer and inexpensive, take a short amount of time to collect the information, and can provide immediate feedback at the point of care and detect potential factors that influence adherence [14]. Artificial intelligence (AI) systems are increasingly being used in health care, with the expectation that such systems will help to develop and increase the capacity of humans in such areas as diagnostics, therapeutics, and the management of patient care and health care systems [15]. AI can provide decisional support to health care providers and patients. Machine learning (ML), which is a subfield of AI, is based on the use of computer algorithms that are able to automatically improve through experience. ML has been extensively applied to the biomedical domain, in particular, using data retrieved from large medico-administrative databases of hospital data warehouses. However, a PROM to assess medication adherence based on ML has not yet been developed. Combining objective measures with patient-reported measures can improve the ability of ML algorithms to assess the health status of patients and improve the delivery of health care [16]. We hypothesized that an ML approach could be used to develop tools, such as decision trees, to model the complexity and possible interactions between patient behaviors and beliefs that lead to medication adherence. Indeed, there are sometimes discrepancies between what patients say to their doctor and their actual behavior concerning their treatment. Decision trees that are created using a supervised ML approach can model a patient’s medication adherence from data obtained from a PROM [17].

We aimed to create and validate an easy-to-use PROM on medication adherence interpreted with a decision tree and easily implementable in computerized prescriber order-entry systems and digital tools.

## Methods

### Overview

The results of the study are reported according to the Standards for Reporting of Diagnostic Accuracy Studies guidelines [18].

### Initial Item-Set Building Using the Delphi Process

The consensus in the choice of the item wording of the questionnaire was compiled following the discussions using the Delphi process during May 2020 to confront and converge the thoughts and opinions of the expert panel, with the objective of coming to a group consensus [19,20]. Experts were identified by their long-standing activity in medication adherence management. Among the 16 French-speaking experts invited to participate, 15 (11 French, 2 Swiss, 1 Belgian, and 1 Canadian) responded to the first-round questionnaire and then completed the second round. The Delphi panel included 10 physicians (9 hospital practitioners and 1 community practitioner), 3 pharmacists (2 hospital pharmacists and 1 community pharmacist), and 2 patient-association heads. A web-based software (Google Form) was used to host the questionnaires and responses. The first questionnaire consisted of 11 items that encompassed the four dimensions of medication adherence as defined by the WHO [2], which are the (1) disease, (2) patient, (3) treatment, and (4) physician-patient relationship. Four different wordings were suggested for each item. Based on a narrative literature review and our experience in PROM validation [18], 2 core group members (VKS and BS) designed the first questionnaire and an executive committee (AA and GP) approved it. VKS, BS, AA, and GP did not respond to the various rounds of the Delphi process. Experts had to independently rate each question related to an item using a 4-point scale according to its relevance and comprehensiveness. Experts were invited for the first-round questionnaire by email and 2 reminders were sent within 2 weeks. Respondents were included in the consensus process and participated in the subsequent 2 rounds. The responses and comments remained anonymous, except for the moderators.

### Setting and Study Design

For internal validation of the PROM, we conducted an observational, cross-sectional, single-center study in the European Georges Pompidou Hospital (GHU Paris Centre, APHP), a 751-bed teaching hospital in Paris, France. With the aim of building a generalizable medication adherence tool, consecutive patients attending the outpatient hypertension, nephrology, oncology, pneumology, and HIV units between March 2021 and March 2022 were invited to participate. To be included in the study, patients had to (1) be receiving at least 1 long-term treatment, (2) be able to read or understand French, (3) be older than 18 years, (4) provide their nonopposition consent, and (5) have a medication adherence evaluation other than a PROM.

### Data Collection

Data were collected in an electronic case report form (Research Electronic Data Capture [REDCap], Vanderbilt University) [21] including (1) the responses to the initial questionnaire at inclusion, (2) sociodemographic characteristics, and (3) a comparative medication adherence assessment extracted from patient charts. The comparative medication adherence evaluations considered were the standard medication adherence assessments used in the daily practice of each outpatient unit: therapeutic drug monitoring for immunosuppressants and oral anticancer drugs, urinary screening for antihypertensive drugs by ultraperformance liquid chromatography or mass spectrometry, routinely performed in our hospital [22], physician evaluation of the level of asthma control, and the medication possession ratio, based on pharmacy refill data for all drugs, when available, generally defined as the proportion of a time period for which a medication supply is available [23,24], in accordance with the chronic disease (Table 1). In terms of clinical data, we did not include blood pressure readings, because they do not depend solely on medication adherence, or CD4 lymphocyte counts to determine the medication adherence of patients with HIV, because in a systematic review published in 2016, the authors concluded that the majority of studies found no difference in the odds of adherence when comparing CD4 lymphocyte count strata [25]. When patients had more than 1 comparative medication adherence measure (this was the case for hypertension, oncology, and kidney transplantation), 4 authors (VKS, YT, AA, and BS) independently and blindly scored medication adherence and a consensus was reached during a meeting. Medication adherence evaluations were further classified into low, medium, or high adherence using the ratings presented in Table 1.

**Table 1:** Ratings of comparative medication adherence evaluations in accordance with the chronic diseases.

Method	Outpatient service	Rating	Reference
Medication possession ratio based on pharmacy refill data	HIV Pneumology Oncology Transplantation Hypertension	Low adherence<50% Medium adherence≥50%<80% High adherence≥80%	Sattler et al [23]
Therapeutic drug monitoring	Oncology Transplantation	Low adherence = result under limit of quantification Medium adherence = subtherapeutic level without clinical or pharmacological explanations High adherence = therapeutic and supratherapeutic ranges	Zijp et al [26]
Urinary screening	Hypertension	Low adherence = negative Medium adherence = traces High adherence = positive	Kably et al [22]
Physician evaluation	Pneumology	Low adherence = very bad and bad Medium adherence = moderate and good High adherence = very good	George [27]

### Item Reduction

From the initial questionnaire, items that were not statistically associated with the medication adherence status by the chi-square test or Fisher exact test (threshold *P*<.1), as evaluated by the standard methods used in each outpatient unit, were removed to obtain the final set of items.

### Development of the Decision Tree

We adopted a traditional approach for the decision tree performance assessment [28]. We split the data set into 3 subsets: the learning data set (containing 130/218, 60% of the patients), the validation data set (44/218, 20% of the patients), and a test data set (the remaining 44/218, 20% of the patients) to avoid overfit. As the data set was unbalanced in terms of distribution of the 3 medication adherence classes, the split was stratified on this factor to maintain the same proportion of each class in each subset. Moreover, this was specified in the hyperparameters by the class weight parameter. We obtained the best set of hyperparameters by 4-fold cross-validation.

Due to coding of the medication adherence into 3 classes, we provided a strict definition (proportion of patients in the diagonal) and a weakened definition for accuracy.

To choose the best hyperparameters, this weak-accuracy formula (Figure S1 in Multimedia Appendix 1) was used in GridSearchCV to maximize the chances of finding poorly adherent and moderately adherent individuals while keeping the maximum number of highly adherent individuals correctly classified.

Two approaches to the answers of the PROM were tested to compare their performance in terms of accuracy. The variables were considered first as qualitative variables and then as continuous variables. The same random seed was applied.

### Evaluation of the PROM Psychometric Properties

We evaluated the sensitivity, specificity, and positive and negative predictive values (PPV and NPV), accompanied by 95% CI, to report the capacity of the decision tree to correctly classify patients according to medication adherence based on the test subset when pooling the patients with low and medium adherence against those with high adherence. Cronbach α score [29] was calculated to check the internal consistency of the dimensions as defined through the Delphi process. A sensitivity analysis was performed and is presented in Table S1 in Multimedia Appendix 1.

### Ethical Considerations

The study was conducted according to good clinical practices for biomedical studies according to French regulations. The study protocol, patient-information note, and nonopposition consent form were approved by an ethics committee (CERAPHP 16/09/20, IRB registration 00011928). The study was explained to all potentially eligible patients. Inclusion was validated after patient nonopposition was received.

### Statistical Analysis

The characteristics at inclusion were compared according to the comparative medication adherence evaluations into 3 classes using standard tests: Student *t* tests or nonparametric Wilcoxon-Mann-Whitney tests, depending on the variable distribution, for continuous parameters and chi-square or exact Fisher tests, depending on the frequency, for qualitative parameters.

Results are presented as means and 1 SD if the parameter follows a Gaussian distribution or, otherwise, as medians for continuous parameters. For qualitative parameters, the results are presented as numbers (%).

All statistical analyses were performed using R software (R Core Team) [30]. The decision tree approach was built using Python 3.8.3 (Python Software Foundation; MSC version 1916 64 bit [AMD64]] with the scikit-learn 1.1.1 library [31] using the DecisionTreeClassifier procedure (Figure S2 in Multimedia Appendix 1). Moreover, the GridSearchCV (sklearn library of Python) procedure [31] allowed finding the best hyperparameters.

## Results

### Item Wording Selection From the Delphi Process

The first-round questionnaire consisted of 11 items that encompassed the four dimensions of medication adherence as defined by the WHO [2], which are the (1) disease, (2) patient, (3) treatment, and (4) physician-patient relationship. Four different wordings were suggested for each item. Experts had to independently rate each question related to an item using a 4-point scale according to relevance and comprehensiveness. After the first round, only 1 item did not reach consensus, thus requiring a second round. The consensus was achieved for the wording of each item at the end of the second round.

**Figure 1:**
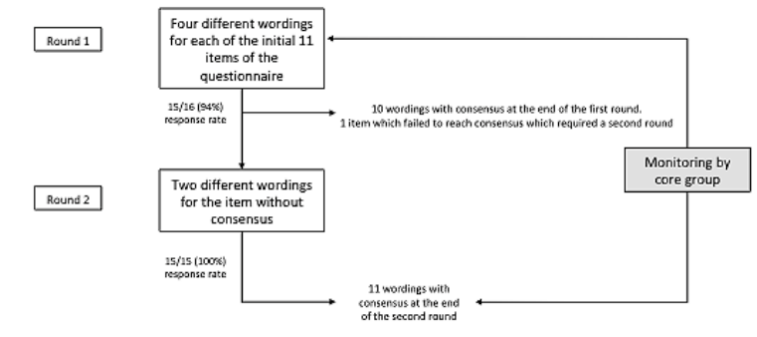
Description of the Delphi process.

**Figure 2:**
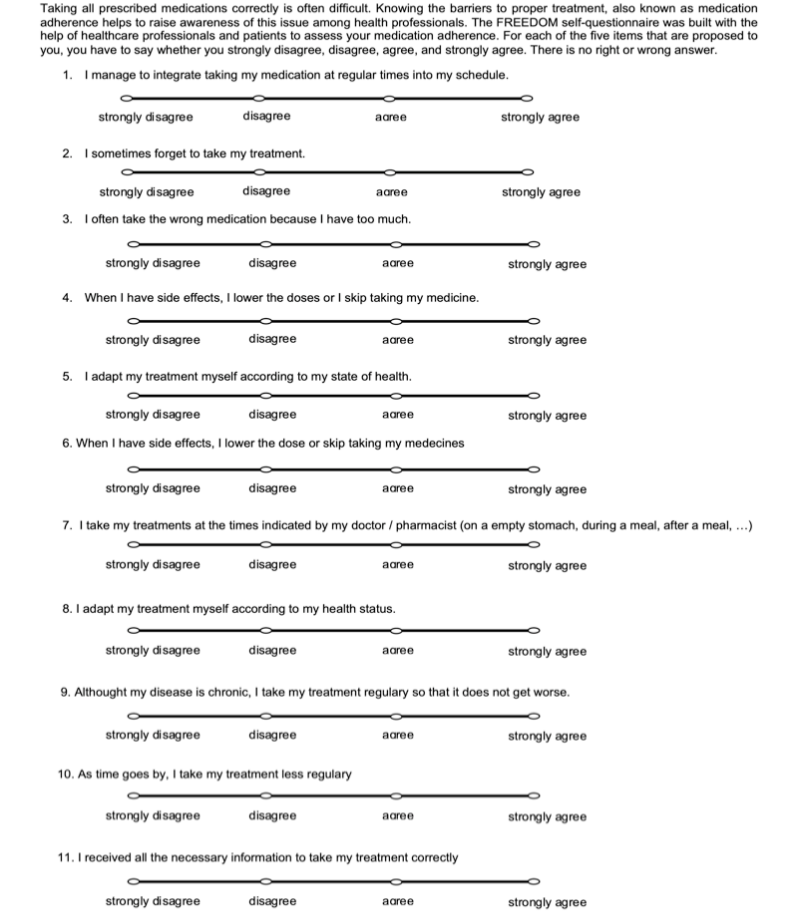
11-items FREEDOM questionnaire. FREEDOM: FREE Detection non Observance Medication.

In round 1, experts had to define the best wording in a series of 4 different wordings for each of the 11 initial items of the questionnaire. Their responses were collected to identify the best (11 items) questionnaire in terms of relevance and comprehensiveness. In round 2, experts were asked to choose the best wording for the single item for which they were unable to reach a consensus at the end of round 1 (Figures 1 and 2).

### Clinical and Demographic Data

**Figure 3:**
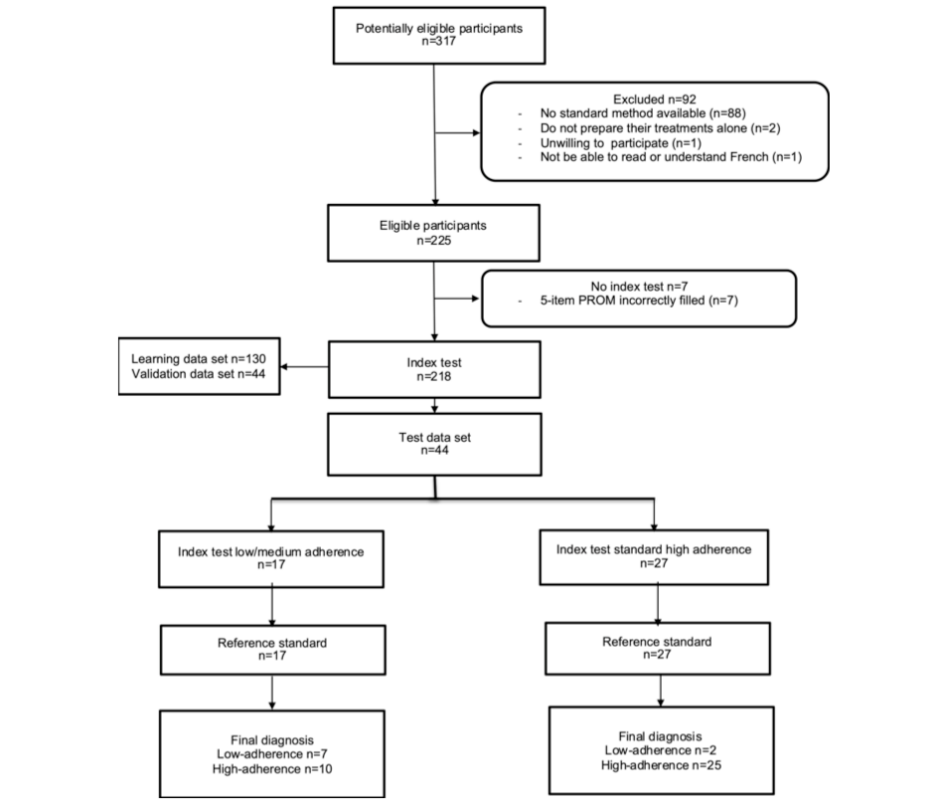
Flowchart of the study.

A flowchart of the study is provided in Figure 3.

The sociodemographic data and medication adherence of the 218 included patients are presented in Table 2. The mean age was 58.1 (SD 4.6) years, and 46.3% (101/218) were women. Chronic diseases were hypertension (n=61, 28%), cancer (n=60, 27.5%), kidney transplantation (n=39, 18%), asthma (n=29, 13.3%), and outpatient dispensing by hospital pharmacy (n=29, 13.3%). The number of medications prescribed per patient was 7.1 (SD 4.7), with an average of 9.1 (SD 6.9) pills prescribed per day. Most patients had recently (<1 year) started their long-term therapy (n=98, 46.5%), with 57 patients (27%) being on their treatment for 10 years or more.

**Table 2:** Sociodemographic characteristics and medication adherence level of the population (N=218).

Demographic variable		Values
Female, n (%)		101 (46.3)
Age (years), mean (SD)		58.1 (14.6)
**Class age (years), mean (SD)**		
	<55	87 (39.9)
	≥55	131 (60.1)
**Disease condition, n (%)**		
	Hypertension	61 (28.0)
	Kidney transplantation	39 (17.9)
	Oncology	60 (27.5)
	Asthma	29 (13.3)
	Outpatients dispensing (HIV and pulmonary arterial hypertension)	29 (13.3)
Number of prescription drugs, mean (SD)		7.1 (4.7)
Number of pills to take, mean (SD)		9.1 (6.9)
**Duration on chronic medication (years), n (%)**		
	<1	98 (45.0)
	<5	35 (16.1)
	<10	21 (9.6)
	≥10	57 (26.1)
	N/A^a^	7 (3.2)
**Medication adherence^b,c^, n (%)**		
	High adherence	171 (78.4)
	Medium adherence	21 (9.6)
	Low adherence	26 (11.9)

^a^N/A: not applicable.

^b^Based on medication possession ratio or drug levels or urinary screening.

^c^26 patients were evaluated by 2 methods.

### Medication Adherence Assessment

Among the 218 patients, 11.9% (26/218) of them were considered to have low medication adherence, 9.6% (21/218) medium medication adherence, and 78.4% (171/218) high medication adherence, based on the results of medication adherence assessment standard used in the daily practice of each outpatient unit. This corresponded to 21.6% (47/218) of patients with suboptimal medication adherence. In bivariate analysis, there was a higher statistically significant risk of low medication adherence associated with younger age (<55 years) and for certain therapeutic classes of antihypertensive medications and for immunosuppressive drugs in kidney transplantation (Table 3).

**Table 3:** Determinants of medication adherence level (N=218).

Demographic variable		High adherence (n=171), n (%)	Medium adherence (n=21), n (%)	Low adherence (n=26), n (%)	*P* value
Female, n (%)		79 (46.2)	7 (33.3)	15 (57.7)	.249
Age (years), mean (SD)		59.7 (14.3)	52.3 (13.2)	52 (14.9)	.006^a^
**Class age (years), mean (SD)**					.002^a^
	<55	58 (34)	14 (67)	15 (57.7)	
	≥55	113 (66.1)	7 (33.3)	11 (42.3)	
**Disease condition, n (%)**					<.001^a^
	Hypertension	37 (21.6)	11 (52.4)	13 (50)	
	Kidney transplantation	36 (21.1)	0 (0)	3 (11.5)	
	Oncology	53 (31)	4 (19.1)	53 (31)	
	Asthma	17 (9.9)	5 (23.8)	7 (26.9)	
	Outpatients dispensing	28 (16.4)	1 (4.8)	0 (0)	
Number of prescription drugs, mean (SD)		7 (4.1)	8.1 (6.4)	7.2 (6.2)	.66
Number of pills to take per day, mean (SD)		9 (5.7)	10.7 (11.7)	8.9 (8.4)	.44
**Duration on chronic medication (years), n (%)**					.69
	<1	78 (46.7)	10 (52.6)	10 (40)	
	<5	29 (17.4)	3 (15.8)	3 (12)	
	<10	16 (9.6)	3 (15.8)	2 (8)	
	≥10	44 (26.4)	3 (15.8)	10 (40)	
**Therapeutic classes, n (%)**					
	β-Blockers	6 (23.1)	7 (33.3)	23 (13.4)	.04^a^
	Diuretics	8 (30.8)	8 (38.1)	22 (12.9)	.003^a^
	Conversion enzyme inhibitors	3 (11.5)	5 (23.8)	19 (11.1)	.27
	Calcium channel blockers	10 (38.5)	9 (42.9)	34 (19.9)	.01^a^
	Sartans	7 (26.9)	4 (19.1)	15 (8.8)	.02^a^
	α-Blockers	4 (15.4)	1 (4.8)	9 (5.3)	.12
	Central antihypertensives	5 (19.2)	2 (9.5)	7 (4.1)	.01^a^
	Antialdosterone	5 (19.2)	1 (4.8)	8 (4.7)	.03^a^
	Tyrosine kinase inhibitors	2 (7.7)	4 (19.1)	33 (19.3)	.37
	Antiretrovirals	0 (0)	1 (4.8)	20 (11.7)	.12
	Poly-ADP ribose polymerase inhibitors	0 (0)	0 (0)	6 (3.5)	>.99
	Vaptan	0 (0)	0 (0)	1 (0.6)	>.99
	Phosphodiesterase-5 inhibitors	0 (0)	0 (0)	2 (1.2)	>.99
	Endothelin antagonists	0 (0)	0 (0)	3 (1.7)	>.99
	Soluble guanylate cyclase	0 (0)	0 (0)	1 (0.6)	>.99
	Immunosuppressants	3 (11.5)	1 (4.8)	39 (22.8)	.08^a^
	Corticosteroids	3 (11.5)	0 (0)	16 (9.4)	.31
	Cyclin-dependent kinase inhibitors	1 (3.8)	0 (0)	5 (2.9)	.77
	Spindle poisons	0 (0)	0 (0)	1 (0.6)	>.99
	Antipyrimidines	0 (0)	0 (0)	3 (1.7)	>.99

^a^*P*<.10.

### Item Reduction

**Figure 4:**
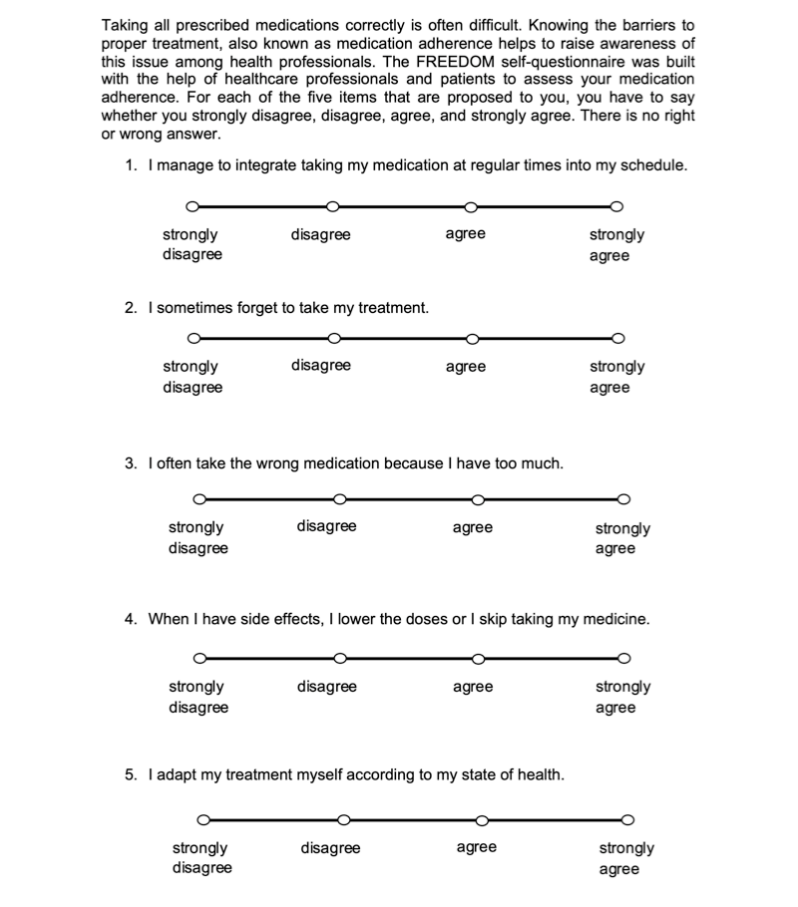
5-item FREEDOM questionnaire. FREEDOM: FREE Detection non Observance Medication.

The patients’ responses to the initial 11-item questionnaire depending on their medication adherence level are presented in Table 4. Among the original 11 items, 5 (#3, #4, #5, #6, and #8) correlated with the 3 classes of the patients’ adherence level as determined by the medication adherence assessment standard used in the daily practice of each outpatient unit (*P* value<.1) and were then integrated into the decision tree model (Figure 4).

**Table 4:** Responses of included patients to the FREEDOM^a^ questionnaire depending on their medication adherence level.

Items and responses			Population (N=218), n (%)	Low adherent patients (n=26), n (%)	Medium adherent patients (n=21), n (%)	High adherent patients (n=171), n (%)	*P* value
**Dimension of medication adherence: the patient**							
	**(1) I am suspicious of medication**						.31
		Strongly disagree	104 (47.7)	15 (57.7)	9 (42.9)	80 (46.8)	
		Disagree	36 (16.5)	1 (3.8)	3 (14.3)	32 (18.7)	
		Agree	55 (25.2)	5 (19.2)	7 (33.3)	43 (25.1)	
		Strongly agree	23 (10.5)	5 (19.2)	2 (9.5)	16 (9.4)	
	**(2) My entourage is very involved in the management of my disease, and ensures that I take my treatment correctly**						.13
		Strongly disagree	51 (23.5)	2 (7.7)	8 (38.1)	41 (24.1)	
		Disagree	24 (11.1)	5 (19.2)	0 (0)	19 (11.2)	
		Agree	45 (20.7)	7 (26.9)	4 (19)	34 (20)	
		Strongly agree	97 (44.7)	12 (46.1)	9 (42.9)	76 (44.7)	
		N/A^b^	1 (0)	0 (0)	0 (0)	1 (0)	
	**(3) I manage to integrate taking my medication at regular times into my schedule**						.003^c^
		Strongly disagree	7 (3.2)	2 (7.7)	2 (9.5)	3 (1.7)	
		Disagree	17 (7.8)	5 (19.2)	4 (19)	8 (4.7)	
		Agree	57 (26.1)	8 (30.8)	4 (19)	45 (26.3)	
		Strongly agree	137 (62.8)	11 (42.3)	11 (52.4)	115 (67.2)	
	**(4) I sometimes forget to take my treatment**						.001^c^
		Strongly disagree	114 (52.3)	7 (26.9)	8 (38.1)	99 (57.9)	
		Disagree	34 (15.6)	3 (11.5)	3 (14.3)	28 (16.4)	
		Agree	57 (26.1)	11 (42.3)	7 (33.3)	39 (22.8)	
		Strongly agree	13 (6)	5 (19.2)	3 (14.3)	5 (2.9)	
**Dimension of medication adherence: the treatment**							
	**(5) I often take the wrong medication because I have too many to take**						.01^c^
		Strongly disagree	176 (80.7)	18 (69.2)	13 (61.9)	145 (84.8)	
		Disagree	31 (14.2)	7 (26.9)	5 (23.8)	19 (11.1)	
		Agree	8 (3.7)	1 (3.8)	1 (4.8)	6 (3.5)	
		Strongly agree	3 (1.4)	0 (0)	2 (9.5)	1 (0.6)	
	**(6) When I have side effects, I lower the dose or I skip taking my medicine**						.004^c^
		Strongly disagree	163 (74.8)	15 (57.7)	13 (61.9)	135 (78.9)	
		Disagree	24 (11)	3 (11.5)	3 (14.3)	18 (10.5)	
		Agree	19 (8.7)	4 (15.4)	1 (4.8)	14 (8.2)	
		Strongly agree	12 (5.5)	4 (15.4)	4 (19)	4 (2.3)	
	**(7) I take my treatments at the times indicated by my doctor or pharmacist (on an empty stomach, during a meal, after a meal, etc)**						.38
		Strongly disagree	8 (3.7)	2 (7.7)	1 (4.8)	5 (2.9)	
		Disagree	11 (5)	3 (11.5)	1 (4.8)	7 (4.1)	
		Agree	62 (28.4)	7 (26.9)	7 (33.3)	48 (28.1)	
		Strongly agree	137 (62.8)	14 (53.8)	12 (57.1)	111 (64.9)	
**Dimension of medication adherence: the disease**							
	**(8) I adapt my treatment myself according to my state of health**						.07^c^
		Strongly disagree	151 (69.3)	13 (50)	14 (66.7)	124 (72.5)	
		Disagree	33 (15.1)	4 (15.4)	4 (19)	25 (14.6)	
		Agree	24 (11)	7 (26.9)	1 (4.8)	16 (9.4)	
		Strongly agree	10 (4.6)	2 (7.7)	2 (9.5)	6 (3.5)	
	**(9) Although my disease is chronic, I take my treatment regularly so that it does not get worse**						.74
		Strongly disagree	8 (3.7)	1 (3.8)	1 (4.8)	6 (3.5)	
		Disagree	3 (1.4)	1 (3.8)	0 (0)	2 (1.2)	
		Agree	39 (18)	4 (15.4)	5 (23.8)	30 (17.6)	
		Strongly agree	167 (77)	20 (76.9)	15 (71.4)	132 (77.6)	
		N/A	1 (0)	0 (0)	0 (0)	1 (0)	
	**(10) Over time, I take my treatment less regularly**						.86
		Strongly disagree	171 (78.8)	19 (73.1)	17 (80.9)	135 (79.4)	
		Disagree	28 (12.9)	4 (15.4)	3 (14.3)	21 (12.3)	
		Agree	10 (4.6)	2 (7.7)	0 (0)	8 (4.7)	
		Strongly agree	8 (3.7)	1 (3.8)	1 (4.8)	6 (3.5)	
		N/A	1 (0)	0	0	1 (0)	
**Dimension of medication adherence: the physician-patient relationship**							
	**(11) I received all the necessary information to take my treatment correctly**						.45
		Strongly disagree	6 (2.8)	2 (7.7)	0 (0)	4 (2.3)	
		Disagree	5 (2.3)	0 (0)	1 (4.8)	4 (2.3)	
		Agree	40 (18.4)	4 (15.4)	2 (9.5)	34 (20)	
		Strongly agree	166 (76.5)	20 (76.9)	18 (85.7)	128 (75.3)	
		N/A	1 (0)	0 (0)	0 (0)	1 (0)	

^a^FREEDOM: FREE Detection non Observance Medication.

^b^N/A: not applicable.

^c^*P*<.10.

### Development of the Decision Tree

**Figure 5:**
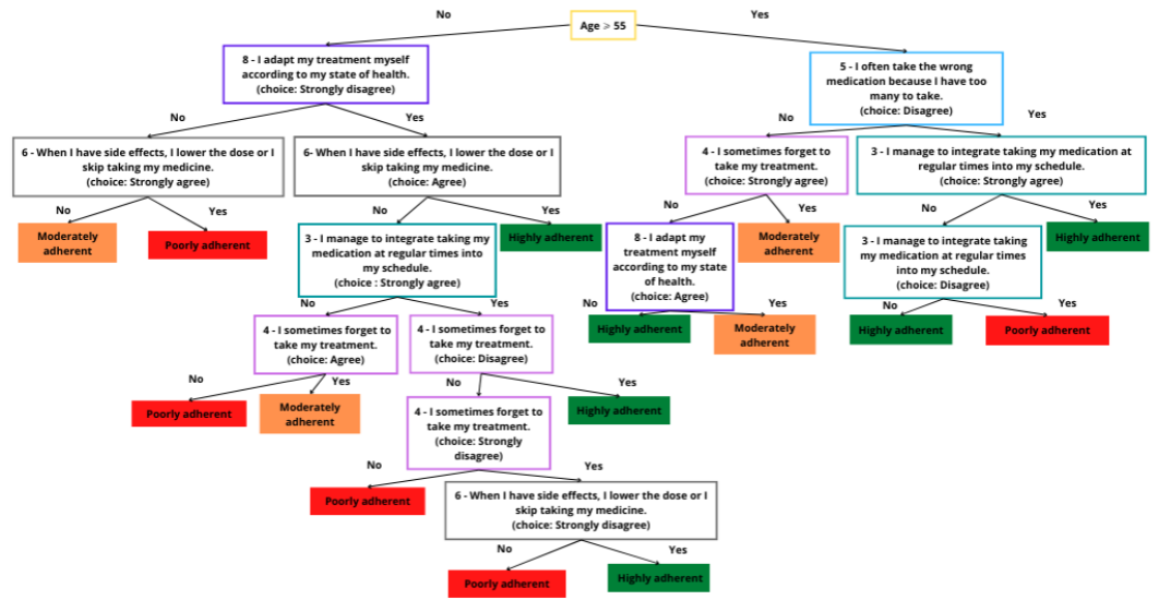
Decision tree to predict patients’ medication adherence.

The final decision tree is presented in Figure 5. All disease groups were included in the training, validation, and test data sets, and we paid attention to have the same proportion of patients classified as nonadherent, moderately adherent, and adherent in each data set. The baseline characteristics in each data set are presented in Table S2 in Multimedia Appendix 1). It was composed of 14 binary decision nodes and had a depth of 7 levels. The test data subset enabled assessment of the 2 approaches (qualitative or continuous) concerning the 4-point Likert-scaled answers to the PROM questions in terms of accuracy and the weak definition of accuracy. Regardless of the approach, the order of magnitude of the performance did not change (±5%). We favored the approach using the answers as qualitative variables, as it would be easier to apply for a doctor handling a paper questionnaire with single-choice questions.

### Evaluation of the PROM Psychometric Properties

For internal consistency, we determined the Cronbach coefficient for the patient dimension (#1 and #2; =.54; 95% CI 0.39-0.65) and the treatment dimension (#3 and #4; =.60; 95% CI 0.41-0.74). The Cronbach coefficient was not determined for the disease dimension because only 1 item concerned this dimension in the final 5-item PROM.

The accuracy of the decision trees with the strict and weakened definitions was 70% (95% CI 55%-83%) and 82% (95% CI 72%-89%), respectively. After pooling the patients with low and medium adherence against those with high adherence, the decision tree led to a sensitivity of 78% (95% CI 40%-96%), a specificity of 71% (95% CI 53%-85%), a PPV of 41% (95% CI 19%-67%), an NPV of 93% (95% CI 74%-99%), and an accuracy of 70% (95% CI 55%-83%).

## Discussion

This study reports the creation and internal validation of a new PROM on medication adherence, interpreted using an ML approach based on a decision tree, which showed good psychometric properties. This is the first PROM including 5 items with responses scored on a 4-point Likert scale to study 3 dimensions of medication adherence (the patient, treatment, and disease). Decision trees are a reliable and effective decision-making technique that provides high classification accuracy with a simple representation of gathered knowledge [30,31] and have been used in various areas of medical decision-making [12,13]. In the medication adherence area, decision trees allow modeling of the complexity of adherence‐related behaviors of patients. Indeed, there are often discrepancies between what patients say and their actual behavior [32].

The Delphi process ensured the relevance and comprehensiveness of the initial 11 items obtained by consensus with a large panel of French-speaking experts. The association test between item responses and the results of standard medication adherence assessment used in the daily practice of each outpatient unit ensured that the final 5 selected items are reliable hallmarks for medication adherence. This methodology enhanced the appropriateness of the questions asked of the patients.

Our tool showed good psychometric properties. In theory, for the internal consistency measurement property, a Cronbach α≥.70 for each unidimensional scale or subscale is considered to be very good [21]. It is difficult to compare individual medication adherence PROM proprieties because of the diversity of the studied populations, the difference in the elements of comparison, and the heterogeneously and empirically fixed cutoff of nonadherence depending on the study. The PPV of our tool is modest (41%, 7/17) but this is likely related to the limited number of nonadherent patients included (n=26). This point will be improved during the step of external validation on a larger number of subjects, with an effort to include more patients with diseases associated with low adherence, for example, asthma, to improve the performance and psychometric parameters of the tool, which will improve the tool’s proprieties. However, the NPV (93%, 25/27) was good, which is valuable in clinical practice: patients with a negative result, which is considered to indicate adherence, will not be wrongly addressed to more intensive and time-consuming medication adherence interventions. However, the tool showed an excellent NPV, which can be considered as an operational advantage, as it could allow prioritization to avoid time- and resource-consuming interventions that aim to reinforce medication adherence for patients who are already highly adherent (Figure 3).

The initial 11-item PROM was performed at 2 time points for a significant proportion of patients included in our study because we aimed to investigate whether our tool was able to detect and quantify any improvement in medication adherence. We did not study classical psychometric parameters, such as test-retest, to evaluate intra-assessor reliability, as we considered that such metrics would be biased in our context. Indeed, patients received educational interventions that aimed to reinforce medication adherence during the hospital appointment during which the PROM was completed. Thus, the results of an initial PROM collection are not expected to be the same as those from a PROM collection at a later time point, which could artificially lead to poor test-retest performance.

There is no consensus on the best method to evaluate medication adherence, but PROMs have demonstrated good results [29,33] and can be used to study the reasons for nonadherence. The biases of PROMs are well known, such as the acquiescent bias, perception bias, or Hawthorne effect [34]. To limit these biases, we focused on the wording of the questions and proposed responses on a Likert scale [35]. Indeed, we hypothesized that medication adherence, which is a complex and multifactorial behavior, cannot be satisfactorily assessed using a dichotomous approach. We reasoned that the Likert scale allows patients to nuance their responses and using pair propositions forces the respondents to take a stand, as there is no neutral answer [35].

In the interest of representativeness, we chose to include patients with different diseases in terms of symptoms and prognoses, such as hypertension, which is generally asymptomatic, or cancer, which is associated with prognostic challenges. Nearly half of the included patients (45.9%, 100/218) had diseases engaging their short-term vital prognosis, with immunosuppressive drugs for kidney transplantation and oral antitumoral drugs for cancer, which may explain the higher level of adherence of our included patients (78.4%, 171/218) than those included in other studies (50%) [36]. The large number of patients that recently started chronic treatment could explain the small number of included low-adherence patients [37]. As previously described in the literature, we found that young age is a risk factor of nonadherence [38]; our cutoff was 55 years. We also found that the drugs with the most adverse effects were also associated with a risk of nonadherence, as in most studies [39-41]. In the context of the French health care system, studying insurance coverage was not considered relevant because all patients necessarily have national health insurance and full refunding of health care expenditures associated with the chronic conditions studied. Although of great interest, we were unable to study racial and ethnic information, as French legislation does not allow the recording of race or ethnicity in the health care system (CNIL, art 9 RGPD) or the collection of such information for research.

The main limitations of our study were the small number of patients included and the single-center design, which are, however, considered sufficient for internal validation [29]. Moreover, we did not collect data on socioeconomic status, racial and ethnic status, or insurance coverage, although these factors are recognized to influence medication adherence [42].

Another limitation was that we compared our 5-item questionnaire to different methods of medication adherence evaluation as gold standards [43]. We mixed the results of the medication adherence assessment standards used in the daily practice of each outpatient unit.

In conclusion, our 5-item questionnaire shows a number of qualities for clinical practice and research activities. First, it shows good psychometric properties with 5 items and is easily and rapidly completed by patients (<5 min). Second, the patients’ responses make it possible to know the reasons for medication nonadherence and then to propose adherence interventions specific to the difficulties met by the patients, such as the reduction, whenever possible, in the number of drugs in the medical prescription, the prevention of adverse effects, a motivational interview, etc. However, the most innovative quality of the present PROM is the ML approach, based on a decision tree derived from data obtained with a 5-item PROM for classifying patients as poorly, moderately, or highly adherent, with an accuracy of 70% and an NPV of 93%, that can be easily implemented in both hospital information and digital tools. For patients, this PROM questionnaire was easy to complete in less than 5 minutes. For clinicians, the tool is easy to use because of its interpretation with a decision tree, which can be easily implemented in both computerized prescriber order entry and digital tools, such as smartphones or e-medicine platforms. At each medical visit, physicians can enter the 5 responses of the patient and immediately obtain his or her medication adherence level. External validation of the tool in a study including a larger number of patients, particularly nonadherent patients, from other medical settings is ongoing and should ill improve the performance of the decision tree. The questionnaire has been translated into English and its validation is also ongoing to make it freely available to the entire medical community.

## Appendix

Multimedia Appendix 1 Index to select the best hyperparameters of the decision tree model.

## Abbreviations

AI: artificial intelligence
ML: machine learning
NPV: negative predictive values
PPV: positive predictive values
PROM: patient-reported outcome measure
REDCap: Research Electronic Data Capture
WHO: World Health Organization

## Data Availability

All data generated or analyzed during this study are included in this published study and in Multimedia Appendix 1.
