# Supplementary material for: Psychometric Properties of a Machine Learning–Based Patient-Reported Outcome Measure on Medication Adherence: Single-Center, Cross-Sectional, Observational Study
Source: J Med Internet Res. 2023 Oct 16;25:e42384. doi: 10.2196/42384 (PMC10616746; doi:10.2196/42384)
Supplement: Multimedia Appendix 1 [file jmir_v25i1e42384_app1.docx]

|  | **Article** | **Tree 1** | **Tree 2** | **Tree 3** | **Tree 4** | **Tree 5** | **Mean** | **SD** | **CV %** |
| --- | --- | --- | --- | --- | --- | --- | --- | --- | --- |
| **Accuracy (matrix 3x3)** | 70.5 | 61.4 | 63.6 | 40.9 | 47.7 | 52.3 | 56.1 | 11 | 19.6 |
| **Accuracy weak (matrix 2x2)** | 85.5 | 82.7 | 84.3 | 67.1 | 73.7 | 76.0 | 78.2 | 7.2 | 9.2 |
| **Sensitivity (matrix 2x2)** | 77.8 | 77.8 | 88.9 | 55.6 | 88.9 | 55.6 | 74.1 | 15.2 | 20.5 |
| **Specificity (matrix 2x2)** | 71.4 | 65.7 | 65.7 | 45.7 | 45.7 | 60.0 | 59.0 | 10.9 | 18.5 |
| **PPV (matrix 2x2)** | 41.2 | 36.8 | 40.0 | 20.8 | 29.6 | 26.3 | 32.5 | 8.2 | 25.2 |
| **NPV (matrix 2x2)** | 92.6 | 92.0 | 95.8 | 80.0 | 94.1 | 84.0 | 89.8 | 6.3 | 7 |
| **DOR (matrix 2x2)** | 8.8 | 6.7 | 15.3 | 1.1 | 6.7 | 1.9 | 6.8 | 5.2 | 76.5 |

**Table S1.** Sensibility analysis.


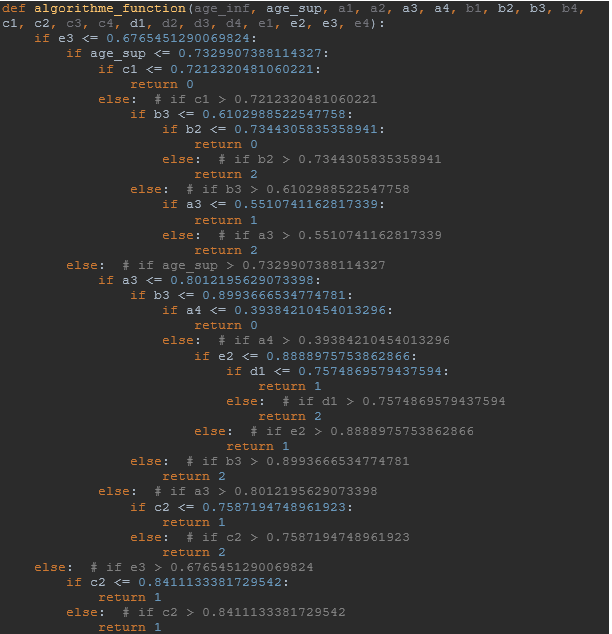


**Figure S2.** Decision tree algorithm**.**

| Variable | Modality | Observant | Mi-observant | Non observant | Echantillon entier | p-value |
| --- | --- | --- | --- | --- | --- | --- |
| Full dataset |  | (N=171) | (N=21) | (N=26) | (N=218) |  |
| clinical_unit | asthme | 17 (9.94%) | 5 (23.81%) | 7 (26.92%) | 29 (13.3%) | < .001 |
|  | HTA | 37 (21.64%) | 11 (52.38%) | 13 (50%) | 61 (27.98%) |  |
|  | oncologie | 53 (30.99%) | 4 (19.05%) | 3 (11.54%) | 60 (27.52%) |  |
|  | rétrocession | 28 (16.37%) | 1 (4.76%) | 0 (0%) | 29 (13.3%) |  |
|  | transplantation rénale | 36 (21.05%) | 0 (0%) | 3 (11.54%) | 39 (17.89%) |  |
| sex | F | 79 (46.2%) | 7 (33.33%) | 15 (57.69%) | 101 (46.33%) | .249 |
|  | M | 92 (53.8%) | 14 (66.67%) | 11 (42.31%) | 117 (53.67%) |  |
| age |  | 59.72 (14.32) 61 [52;71.5] (19;86) | 52.33 (13.16) 52 [45;58] (29;89) | 51.96 (14.87) 53 [48;61] (19;81) | 58.08 (14.55) 58 [50;70] (19;89) | .002 |
| class_age | <55 | 58 (33.92%) | 14 (66.67%) | 15 (57.69%) | 87 (39.91%) | .002 |
|  | >=55 | 113 (66.08%) | 7 (33.33%) | 11 (42.31%) | 131 (60.09%) |  |
| drugs_number |  | 6.99 (4.13) 7 [3.5;10] (1;22) | 8.05 (6.39) 6 [4;10] (1;25) | 7.19 (6.24) 5.5 [2;8.75] (1;20) | 7.12 (4.66) 7 [3;10] (1;25) | .657 |
| prise_number |  | 8.99 (5.74) 9 [4;12] (1;26) | 10.71 (11.71) 7 [5;12] (1;52) | 8.85 (8.37) 6.5 [2;10] (1;28) | 9.14 (6.87) 8 [4;12] (1;52) | .437 |
|  | NA | 5 | 0 | 0 | 5 |  |
| treatment_seniority | < 10 ans | 16 (9.58%) | 3 (15.79%) | 2 (8%) | 21 (9.95%) | .688 |
|  | < 1an | 78 (46.71%) | 10 (52.63%) | 10 (40%) | 98 (46.45%) |  |
|  | < 5 ans | 29 (17.37%) | 3 (15.79%) | 3 (12%) | 35 (16.59%) |  |
|  | > ou = 10ans | 44 (26.35%) | 3 (15.79%) | 10 (40%) | 57 (27.01%) |  |
|  | NA | 4 | 2 | 1 | 7 |  |
| observance | mi-observant | 0 (0%) | 21 (100%) | 0 (0%) | 21 (9.63%) | < .001 |
|  | non observant | 0 (0%) | 0 (0%) | 26 (100%) | 26 (11.93%) |  |
|  | observant | 171 (100%) | 0 (0%) | 0 (0%) | 171 (78.44%) |  |
| Train dataset |  | (N=101) | (N=13) | (N=16) | (N=130) |  |
| clinical_unit | asthme | 10 (9.9%) | 4 (30.77%) | 2 (12.5%) | 16 (12.31%) | < .001 |
|  | HTA | 20 (19.8%) | 7 (53.85%) | 9 (56.25%) | 36 (27.69%) |  |
|  | oncologie | 31 (30.69%) | 1 (7.69%) | 2 (12.5%) | 34 (26.15%) |  |
|  | rétrocession | 14 (13.86%) | 1 (7.69%) | 0 (0%) | 15 (11.54%) |  |
|  | transplantation rénale | 26 (25.74%) | 0 (0%) | 3 (18.75%) | 29 (22.31%) |  |
| sex | F | 45 (44.55%) | 4 (30.77%) | 8 (50%) | 57 (43.85%) | .612 |
|  | M | 56 (55.45%) | 9 (69.23%) | 8 (50%) | 73 (56.15%) |  |
| age |  | 59.46 (15.1) 61 [51;72] (19;86) | 48.92 (11.48) 52 [38;54] (29;72) | 50 (13.32) 53 [47;56.5] (19;67) | 57.24 (15.06) 58 [50;70] (19;86) | .003 |
| class_age | <55 | 36 (35.64%) | 10 (76.92%) | 10 (62.5%) | 56 (43.08%) | .005 |
|  | >=55 | 65 (64.36%) | 3 (23.08%) | 6 (37.5%) | 74 (56.92%) |  |
| drugs_number |  | 7.08 (4.25) 7 [3;10] (1;22) | 6.31 (5.33) 5 [3;7] (1;21) | 9.06 (7.01) 6.5 [3.75;16.25] (1;20) | 7.25 (4.79) 7 [3;10] (1;22) | .529 |
| prise_number |  | 9.15 (5.76) 9 [4;12.5] (1;26) | 7.69 (7.57) 5 [3;9] (1;30) | 11.38 (9.61) 7 [3;20.25] (1;28) | 9.28 (6.54) 8 [4;12.25] (1;30) | .427 |
|  | NA | 2 | 0 | 0 | 2 |  |
| treatment_seniority | < 10 ans | 9 (9.09%) | 2 (16.67%) | 1 (6.67%) | 12 (9.52%) | .311 |
|  | < 1an | 47 (47.47%) | 6 (50%) | 6 (40%) | 59 (46.83%) |  |
|  | < 5 ans | 15 (15.15%) | 2 (16.67%) | 0 (0%) | 17 (13.49%) |  |
|  | > ou = 10ans | 28 (28.28%) | 2 (16.67%) | 8 (53.33%) | 38 (30.16%) |  |
|  | NA | 2 | 1 | 1 | 4 |  |
| observance | mi-observant | 0 (0%) | 13 (100%) | 0 (0%) | 13 (10%) | < .001 |
|  | non observant | 0 (0%) | 0 (0%) | 16 (100%) | 16 (12.31%) |  |
|  | observant | 101 (100%) | 0 (0%) | 0 (0%) | 101 (77.69%) |  |
| Valide dataset |  | (N=35) | (N=4) | (N=5) | (N=44) |  |
| clinical_unit | asthme | 4 (11.43%) | 1 (25%) | 2 (40%) | 7 (15.91%) | .098 |
|  | HTA | 6 (17.14%) | 2 (50%) | 3 (60%) | 11 (25%) |  |
|  | oncologie | 11 (31.43%) | 1 (25%) | 0 (0%) | 12 (27.27%) |  |
|  | rétrocession | 9 (25.71%) | 0 (0%) | 0 (0%) | 9 (20.45%) |  |
|  | transplantation rénale | 5 (14.29%) | 0 (0%) | 0 (0%) | 5 (11.36%) |  |
| sex | F | 17 (48.57%) | 2 (50%) | 3 (60%) | 22 (50%) | 1 |
|  | M | 18 (51.43%) | 2 (50%) | 2 (40%) | 22 (50%) |  |
| age |  | 60.4 (13.77) 60 [53.5;71.5] (20;84) | 59.5 (20.17) 52.5 [48.5;63.5] (44;89) | 50.2 (20.29) 53 [32;66] (27;73) | 59.16 (15.08) 59 [52.25;71.25] (20;89) | .457 |
| class_age | <55 | 11 (31.43%) | 2 (50%) | 3 (60%) | 16 (36.36%) | .44 |
|  | >=55 | 24 (68.57%) | 2 (50%) | 2 (40%) | 28 (63.64%) |  |
| drugs_number |  | 7.14 (3.74) 7 [4;10] (1;14) | 6 (1.63) 6 [5.5;6.5] (4;8) | 4.4 (3.78) 2 [2;8] (1;9) | 6.73 (3.66) 7 [4;9] (1;14) | .302 |
| prise_number |  | 9.19 (5.82) 9 [4.75;13.5] (1;21) | 6.5 (1.73) 6 [5.75;6.75] (5;9) | 4.8 (4.32) 2 [2;9] (1;10) | 8.39 (5.55) 7 [4;11] (1;21) | .241 |
|  | NA | 3 | 0 | 0 | 3 |  |
| treatment_seniority | < 10 ans | 4 (11.76%) | 1 (33.33%) | 0 (0%) | 5 (11.9%) | .621 |
|  | < 1an | 17 (50%) | 2 (66.67%) | 3 (60%) | 22 (52.38%) |  |
|  | < 5 ans | 7 (20.59%) | 0 (0%) | 0 (0%) | 7 (16.67%) |  |
|  | > ou = 10ans | 6 (17.65%) | 0 (0%) | 2 (40%) | 8 (19.05%) |  |
|  | NA | 1 | 1 | 0 | 2 |  |
| observance | mi-observant | 0 (0%) | 4 (100%) | 0 (0%) | 4 (9.09%) | < .001 |
|  | non observant | 0 (0%) | 0 (0%) | 5 (100%) | 5 (11.36%) |  |
|  | observant | 35 (100%) | 0 (0%) | 0 (0%) | 35 (79.55%) |  |
| Test dataset |  | (N=35) | (N=4) | (N=5) | (N=44) |  |
| clinical_unit | asthme | 3 (8.57%) | 0 (0%) | 3 (60%) | 6 (13.64%) | .31 |
|  | HTA | 11 (31.43%) | 2 (50%) | 1 (20%) | 14 (31.82%) |  |
|  | oncologie | 11 (31.43%) | 2 (50%) | 1 (20%) | 14 (31.82%) |  |
|  | rétrocession | 5 (14.29%) | 0 (0%) | 0 (0%) | 5 (11.36%) |  |
|  | transplantation rénale | 5 (14.29%) | 0 (0%) | 0 (0%) | 5 (11.36%) |  |
| sex | F | 17 (48.57%) | 1 (25%) | 4 (80%) | 22 (50%) | .299 |
|  | M | 18 (51.43%) | 3 (75%) | 1 (20%) | 22 (50%) |  |
| age |  | 59.8 (12.8) 61 [53.5;67.5] (29;82) | 56.25 (9) 56 [48.75;63.5] (48;65) | 60 (14.3) 55 [48;68] (48;81) | 59.5 (12.46) 61 [51.75;67.25] (29;82) | .774 |
| class_age | <55 | 11 (31.43%) | 2 (50%) | 2 (40%) | 15 (34.09%) | .726 |
|  | >=55 | 24 (68.57%) | 2 (50%) | 3 (60%) | 29 (65.91%) |  |
| drugs_number |  | 6.6 (4.24) 6 [3.5;8.5] (1;20) | 15.75 (7.63) 14.5 [9.75;20.5] (9;25) | 4 (2.83) 5 [1;6] (1;7) | 7.14 (5.22) 6 [3.75;9] (1;25) | .009 |
| prise_number |  | 8.34 (5.75) 7 [4;11.5] (1;26) | 24.75 (18.89) 17.5 [12;30.25] (12;52) | 4.8 (2.59) 6 [2;7] (2;7) | 9.43 (8.77) 7 [4;12] (1;52) | .01 |
| treatment_seniority | < 10 ans | 3 (8.82%) | 0 (0%) | 1 (20%) | 4 (9.3%) | .42 |
|  | < 1an | 14 (41.18%) | 2 (50%) | 1 (20%) | 17 (39.53%) |  |
|  | < 5 ans | 7 (20.59%) | 1 (25%) | 3 (60%) | 11 (25.58%) |  |
|  | > ou = 10ans | 10 (29.41%) | 1 (25%) | 0 (0%) | 11 (25.58%) |  |
|  | NA | 1 | 0 | 0 | 1 |  |
| observance | mi-observant | 0 (0%) | 4 (100%) | 0 (0%) | 4 (9.09%) | <.001 |
|  | non observant | 0 (0%) | 0 (0%) | 5 (100%) | 5 (11.36%) |  |
|  | observant | 35 (100%) | 0 (0%) | 0 (0%) | 35 (79.55%) |  |

**Table S2.** Baseline characteristics in each dataset (full/train/validation/test dataset).
